# Well-Being at the University: The Contribution of Social and Emotional Competence and Self-Care Practices as Seen by Students

**DOI:** 10.3390/bs16071107

**Published:** 2026-07-03

**Authors:** Sofia Oliveira, Ricardo Pacheco, Luís Curral, Alexandra Marques-Pinto

**Affiliations:** 1 Business Research Unit (BRU), ISCTE—Instituto Universitário de Lisboa, Av. das Forças Armadas, 1649-026 Lisbon, Portugal; 2 Faculdade de Psicologia, Universidade de Lisboa, Alameda da Universidade, 1649-013 Lisbon, Portugal; 3 Research Center for Psychological Science (CICPSI), Faculdade de Psicologia, Universidade de Lisboa, Alameda da Universidade, 1649-013 Lisbon, Portugal; lcurral@psicologia.ulisboa.pt (L.C.); ampinto@psicologia.ulisboa.pt (A.M.-P.)

**Keywords:** academic adaptation, academic engagement, higher education students, self-care practices, social and emotional competence, well-being

## Abstract

Transition to higher education represents a critical period marked by academic, emotional, and social challenges that can affect students’ well-being. Although social and emotional competence (SEC) and self-care practices have been identified as protective factors of well-being, there is a gap in understanding how these concepts intersect within higher education. In an exploratory sequential mixed-methods study, we first explored the main challenges perceived by higher education students in adapting to university and which SEC and self-care practices they perceived as most relevant to promoting their personal and academic well-being. Building on these insights, we then investigated the mediating role of self-care practices in the relationship between students’ SEC and their well-being. In the first stage of the study, 16 higher education students (81.3% female, *M* = 22.19 years) participated in semi-structured interviews; additionally, 204 higher education students (77.9% female, *M* = 22.10 years) responded to an online survey. Qualitative findings suggested that the most significant challenges in the adaptation to university were of a social and emotional nature, related to emotional challenges, interpersonal relationships, and personal organization. To overcome these, students primarily valued intrapersonal competencies such as self-awareness and self-regulation. Participants predominantly described using personal self-care practices, focusing on psychological and emotional care. Generalized linear model-based mediation analysis sustained that both personal and academic self-care practices mediated SEC effects on students’ personal well-being. However, only academic self-care practices mediated SEC effects on their academic well-being. Self-regulation competencies had the strongest effect on students’ personal and academic well-being, providing quantitative support for the prominence attributed to this competency by students during the qualitative phase. This research contributes to a strengthened theoretical understanding of the interplay between higher education students’ SEC, self-care practices, and well-being, offering new empirical evidence on how these relate.

## 1. Introduction

### 1.1. Transition and Adaptation to Higher Education

The transition to higher education is a critical developmental period during which students face a complex array of academic, emotional, and social challenges (Campos et al., 2024; Casanova et al., 2020). When entering university, students often experience significant life changes, such as leaving home, managing their own finances, and adapting to teaching methods that demand greater autonomy and provide less frequent feedback (Campos et al., 2024; Casanova et al., 2020). These changes require adjustments across multiple domains, including academic, social, personal-emotional, and institutional. Such adjustments depend on both individual factors (e.g., personality, self-efficacy) and contextual influences (e.g., family support, peer networks, institutional resources) (Purnamasari et al., 2022). From a developmental perspective, this transition coincides with critical stages of psychosocial development, particularly the consolidation of personal identity and the pursuit of meaningful interpersonal relationships (Erikson, 1982). Students must therefore balance academic demands with building supportive social bonds and developing a coherent sense of self. From a transactional perspective, adaptation to higher education may also be understood as an ongoing process of appraisal and coping, whereby students continuously evaluate environmental demands and their available resources to manage them effectively (Lazarus & Folkman, 1984).

In this scenario, initial contact with university is often described as a ‘shock’, characterized by stress, self-doubt, and demotivation (Campos et al., 2024). The severity of this adjustment period can directly impact higher education students’ academic performance, mental health, and overall well-being (Conley & Donahue-Keegan, 2025). Indeed, longitudinal studies have shown that approximately one-third of students exhibit clinical symptoms of depression and anxiety upon entering university, with prevalence rates increasing after the first year (e.g., Duffy et al., 2020). More broadly, research consistently shows that students’ perceptions of their academic experiences are closely linked to their mental health. Positive perceptions are associated with better well-being, while negative perceptions, such as low support and high academic stress, are associated with anxiety, depression, and reduced life satisfaction (e.g., Fonte & Macedo, 2021).

Considering the scope and severity of these challenges, there is a growing recognition that higher education institutions must move beyond reactive approaches to student mental health, adopting proactive, strengths-based strategies that empower students with the necessary skills and resources to flourish. In this context, Social and Emotional Competence (SEC) and self-care practices have emerged as two particularly promising factors that may contribute to students’ well-being and successful adaptation (Conley & Donahue-Keegan, 2025; Zhong & Xie, 2023). However, despite an increasing body of literature stressing the importance of SEC and self-care in higher education, the specific and dynamic interplay between higher education students’ SEC, self-care practices, and well-being remains underexplored. This study, therefore, aimed to address this gap by adopting a two-phase mixed-methods research design. First, we explored the main challenges that students perceived as being associated with university life, and the SEC and self-care practices that they considered to be the most relevant for promoting and sustaining their well-being. We then examined the mediating role of self-care practices in the relationship between SEC and students’ personal and academic well-being.

### 1.2. Social and Emotional Learning in Higher Education

Social and Emotional Learning (SEL), primarily informed by the Emotional Intelligence Theory (Salovey & Mayer, 1990), is defined as the process through which individuals acquire and effectively apply the knowledge, attitudes, and skills necessary to understand and manage emotions, set and achieve positive goals, feel and show empathy, establish and maintain positive relationships, and make responsible decisions (Durlak et al., 2015, 2025). Although SEL was originally developed for primary and secondary education, its relevance in higher education has increasingly been recognized, as institutions share the mission of educating students to become informed, responsible, socially competent, healthy, thoughtful, and contributing citizens (Conley & Donahue-Keegan, 2025; Elmi, 2020). This framework identifies five interrelated domains of SEC: *Self-awareness*—the capacity to recognize one’s own emotions, thoughts and values, and to understand their influence on behavior; *Self-regulation*—the ability to manage emotions, thoughts, and behaviors effectively across situations, including stress management, impulse control, and goal-setting; *Social awareness*—the capacity to understand others’ perspectives and to demonstrate empathy; *Relationship skills*—the ability to establish and maintain healthy relationships, to communicate effectively, and to negotiate conflicts constructively; and *Responsible decision-making*—the ability to make constructive and ethical choices about personal behavior and social interactions (Durlak et al., 2015).

Literature consistently supports the idea that SEL fosters harmonious relationships, social cohesion and inclusion, positive attitudes toward diversity, equity, and social justice, and improves mental health and well-being among children and adolescents (Cefai et al., 2018). Although research and intervention efforts in higher education are more limited (Reinert, 2019), there is evidence that higher levels of SEC among university students are associated with better overall adjustment, academic performance, persistence, and enhanced well-being and mental health (e.g., Conley & Donahue-Keegan, 2025; Elmi, 2020; Simion, 2023). In higher education, SEL strategies have been linked to improved cognitive development, stronger student-teacher relationships, and increased higher education student confidence, ultimately promoting both academic and personal success (Elmi, 2020; Simion, 2023). However, whilst there is some evidence regarding the importance of SEC for college students’ adjustment and well-being, further research is still needed to better understand which specific social and emotional competencies (also known as social and emotional skills) contribute to students’ well-being and how they do so. For example, since forming new social connections is often part of the transition to higher education, interpersonal skills have been described as vital for supporting higher education students (Conley, 2015). Nevertheless, preliminary data on the relationship between SEC and higher education students’ well-being have emphasized the role of self-regulation (Oliveira et al., 2025). Additionally, previous meta-analyses on SEL interventions’ impacts for both young people and adults have found substantial heterogeneity across studies (Cipriano et al., 2023; Durlak et al., 2011; Oliveira et al., 2021), suggesting the presence of additional explanatory variables in the relationship between SEC and well-being.

### 1.3. The Role of Self-Care

Self-care is conceptualized by Lee and Miller (2013) as an intentional and ongoing process of involvement in practices aimed at promoting and sustaining health and well-being. This definition emphasizes proactivity and intentionality, framing effective self-care as a conscious, deliberate, and continuous practice rather than a reactive response to distress (Markin, 2022). The relevance of self-care in higher education has started to be acknowledged, as university students face elevated levels of stress, anxiety, depression, and burnout that can jeopardize their academic trajectory and overall well-being (Laposha & Smallfield, 2022; Zhong & Xie, 2023). In the face of these demands, self-care functions as a protective factor that not only mitigates the impact of stressors and helps regulate negative emotions but also strengthens proactive mental health management (Roxas, 2023; Zhong & Xie, 2023).

Lee and Miller’s (2013) model distinguishes between two interrelated dimensions: personal self-care and professional self-care. In an academic context, the latter is understood as academic self-care. Personal self-care encompasses practices that promote the individual’s holistic health across physical, psychological, emotional, and social domains (Lee & Miller, 2013). Common personal self-care practices reported by students include getting enough sleep, exercising regularly, practicing mindfulness, maintaining positive self-talk, and seeking social connection (Laposha & Smallfield, 2022; Roxas, 2023; Zhong & Xie, 2023). Academic self-care, in turn, refers to strategies that support effective functioning in the role of a student (Lee & Miller, 2013), such as managing time effectively, maintaining an organized study environment, setting boundaries between academic and personal life, seeking supervision and mentoring, and engaging in reflective practice (Bishop et al., 2024; Tram et al., 2025). This distinction acknowledges that, while personal and academic life are mutually influential, they require different approaches to management (Lee & Miller, 2013).

Effective self-care is more than simply engaging in a set of activities; it requires awareness and deliberate effort (Laposha & Smallfield, 2022; Markin, 2022). Although conceptually related to social and emotional competencies, self-care practices represent a partially distinct construct, with areas of conceptual proximity. In particular, domains such as self-regulation, planning, and adaptive behavior may contribute to both constructs, making some degree of overlap theoretically expected (Fitzgerald et al., 2022; Mahfouz & Gordon, 2021). In this study, we differentiate SEC and self-care at a functional level: whereas SEC refers to the knowledge, attitudes, and skills that enable individuals to understand and regulate themselves and their interactions with others, self-care practices refer to the behavioral enactment of actions aimed at maintaining health, well-being, and effective functioning (Martínez et al., 2021). From this perspective, SEC may facilitate engagement in self-care practices by providing the personal resources necessary to recognize needs, regulate behavior, and make adaptive choices, thereby supporting well-being. Thus, self-care is considered an important variable in this dynamic, as the skills associated with SEC may foster engagement in self-care practices, which in turn support the development and maintenance of well-being (Lee & Miller, 2013; Markin, 2022). However, to date, no study has been found in the literature that explicitly examines the relationship between these two variables and their impact on students’ well-being.

### 1.4. Well-Being: Personal and Academic Dimensions

Well-being is a broad and multifaceted concept that has been conceptualized by different theoretical approaches in the literature. In this study, we considered two specific, complementary approaches: personal well-being and academic well-being. Concerning personal well-being, we followed Keyes et al.’s (2002) model of subjective well-being, which defines subjective well-being as optimal psychological and social functioning, characterized by frequent positive emotions and effective performance in multiple life domains. Based on three interdependent dimensions (i.e., emotional, psychological, and social), subjective well-being is viewed as an expression of comprehensive mental health—Complete State Model of Health—rather than a momentary peak of well-being (Keyes et al., 2002). Personal well-being is a key resource and protective factor throughout the academic experience. Consequently, students with high levels of well-being exhibit higher levels of academic engagement and resilience and experience less loneliness, even in high-pressure situations (Bagdžiūnienė et al., 2025; Marques-Pinto et al., 2025; Sullivan & Celebre, 2025). However, a substantial proportion of students, irrespective of psychopathology, exhibit low levels of personal well-being (Ramos-Zaga, 2024; Visser & Law-van Wyk, 2021).

Alongside personal well-being, a second important outcome of the university experience is academic well-being, conceptualized through the construct of engagement by Schaufeli et al. (2002). According to these authors, engagement is a positive and rewarding work-related mental state characterized by vigor, dedication, and absorption (Schaufeli et al., 2002). The interplay of these dimensions allows for a holistic understanding of the student’s positive experience. Engagement in a university setting has been found to be positively correlated with academic performance and satisfaction with both personal and academic life (Amaral & Frick, 2022; Marques-Pinto et al., 2025). Furthermore, engagement acts as a protective factor against psychological distress and school dropout, being strongly and negatively correlated with emotional exhaustion and cynicism (Schaufeli et al., 2002). Like personal well-being, academic well-being is not a static personality trait, but rather a dynamic and malleable psychological state influenced by a complex interaction of personal and contextual factors. In an environment where students face numerous challenges that can threaten their health and well-being, it is crucial to gain a better understanding of the contributing factors to design more effective interventions.

### 1.5. The Present Study

**Figure 1:**
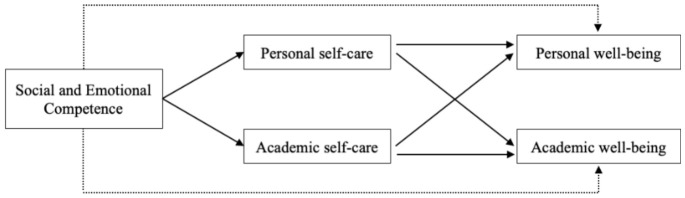
Proposed conceptual model of multiple parallel mediation illustrating the indirect effects of SEC on personal and academic well-being through personal and academic self-care, including cross-domain pathways. *Note.* SEC included five specific skills that were tested individually: Self-awareness, Self-regulation, Positive relationship skills, Conflict management, and Responsible decision making.

Given the above-mentioned gap in the literature, we employed an exploratory sequential mixed-methods design (Creswell & Creswell, 2018). The initial qualitative phase allowed us to explore the challenges, social and emotional competencies, and self-care practices perceived by students as relevant to their well-being. These findings provided contextual grounding for the subsequent quantitative phase, which examined the relationships between higher education students’ SEC, self-care practices, and well-being, including the mediating role of self-care. Furthermore, the qualitative themes informed the interpretation of the quantitative findings, providing contextual insights into the mechanisms underlying the observed associations and mediation effects. Figure 1 depicts the proposed conceptual model. The following research questions guided the study:

*Research Question 1 (RQ1):* What are the main challenges perceived by higher education students in adapting to university?
*Research Question 2 (RQ2):* Which social and emotional competencies do higher education students perceive as most relevant to promoting their personal and academic well-being?
*Research Question 3 (RQ3):* Which self-care practices are considered most important by higher education students to promote their personal and academic well-being?
*Research Question 4 (RQ4):* Do personal and academic self-care practices mediate the relationship between students’ SEC and their personal and academic well-being?

## 2. Materials and Methods

### 2.1. Sample

An initial convenience sample of 16 higher education students (81.3% female, *M* = 22.19 years, *SD* = 2.74) participated in semi-structured interviews. Most students were Portuguese (75.0%), from the University of Lisbon (93.8%), did not hold any special academic status (87.5%), and were enrolled in either an undergraduate (37.5%) or a master’s (62.5%) degree program in Psychology. Regarding personal life, all participants were single or in a non-cohabiting relationship and did not have dependents (e.g., children).

A subsequent convenience sample of 204 higher education students (77.9% female, *M* = 22.10 years, *SD* = 3.58, range: 18–35 years) answered an online survey. Most participants were Portuguese (92.5%), did not hold any special academic status (85.8%), and studied in the same geographical area as their residence (59.3%). Our sample included undergraduate students (63.2%), as well as master’s (29.9%) and PhD students (2.9%). Although a non-probability sampling method was used, our sample included students from all Portuguese counties, providing broad geographic coverage and reducing the likelihood of regional concentration effects. The degree programs attended by the students encompassed a variety of subjects, including Humanities, Arts, Social Sciences (HASS; 72.8%), and Science, Technology, Engineering, and Mathematics (STEM; 27.2%) fields. The HASS programs included Social sciences, journalism and information, Education, Arts and humanities, Business sciences, administration and law, and Services (e.g., psychology, basic education, journalism, design, history, human resources management, marketing, law, social services). STEM programs included Agriculture, forestry, fisheries, and veterinary sciences, Natural sciences, mathematics and statistics, Engineering, manufacturing and construction, Information and communication technologies, and Health and social protection (e.g., veterinary, biology, pharmaceutical sciences, electrical and computer engineering, aeronautic engineering, biostatistics, physics, medicine, nursing). Regarding personal life, most participants were single (58.3%) or in a non-cohabiting relationship (32.4%) and did not have dependents (e.g., children; 96.6%).

### 2.2. Instruments

#### 2.2.1. Qualitative Data

##### Semi-Structured Interview

Semi-structured interviews were conducted using a script specifically developed for the study objectives. The interviews focused on understanding how higher education students perceived their transition and adaptation to university, and which social and emotional competencies and self-care practices they perceived as most relevant to promoting their personal and academic well-being. The script incorporates open-ended questions organized into three main themes: (1) Students’ well-being and adaptation to higher education (e.g., “*What has changed in your life since you started college?*”); (2) Students’ SEC (e.g., “*How has it been to build new relationships at university?*”); and (3) Students’ self-care practices (e.g., “*How do you take care of your emotional/mental well-being?*”). In addition to the three core sets of questions, the interview began with a brief overview of its objectives and procedures and concluded with a concise summary of the main topics. Before concluding the interview, participants were allowed to share any additional comments they deemed relevant. To ensure the script was clear, neutral, and in line with the interviews’ objectives, we followed Creswell’s (2007) and Amado’s (2014) guidelines. Questions were structured from general to specific and were contextualized (e.g., participants were asked to reflect on “their academic experience”, “personal circumstances”, and “university life”). If the participant was unable to elaborate on their answer or focused only on specific aspects, pre-established questions with counter-argument responses based on common and practical scenarios from university life were presented. For example, if the participant focused solely on interpersonal skills, an alternative angle focusing on intrapersonal skills would be introduced, for example: “*Some of your colleagues also mentioned that they had gained a better understanding of themselves since starting university. Have you experienced the same thing?*” The script was carefully constructed to focus only on aspects relevant to the study objectives, respecting the privacy of the participants.

#### 2.2.2. Quantitative Data

##### Social and Emotional Competence

The Social and Emotional Competence Assessment Battery for Adults—Students Survey (SECAB-A(S); Oliveira et al., 2025) was employed to assess students’ use of social and emotional competencies. The SECAB-A(S) includes three independent questionnaires that assess self-awareness (7 items, ω = 0.85), self-regulation (8 items, ω = 0.88), positive relationship skills (8 items, ω = 0.80), conflict management skills (8 items, ω = 0.75), and responsible decision-making (6 items, ω = 0.80). Items (37; e.g., “*During stressful moments at university, I can stay calm.*”) were rated on a 10-point scale (from 1—*Never* to 10—*Always*).

##### Self-Care Practices

Students’ involvement in self-care practices was assessed with the Portuguese version of the Self-care Practices Scale (SCPS; Oliveira et al., in press). The SCPS includes 18 items (e.g., “*I spend quality time with people I care about*.”) that assess how frequently participants engage in personal (9 items, ω = 0.67) and academic (9 items, ω = 0.71) self-care practices, rated on a 5-point Likert scale (from 1—*Never* to 5—*Very often*).

##### Personal Well-Being

The Portuguese adaptation of the Mental Health Continuum—Short Form (MHC-SF; Matos et al., 2010) was used to measure participants’ personal well-being. The MHC-SF includes 14 items (e.g., “*How often have you felt happy?*”; *ω* = 0.91), which were rated considering the frequency of the described feeling in the previous month on a 6-point scale (from 0—*Never* to 5—*Every day*).

##### Academic Well-Being

Academic well-being was measured with the 9-item (*ω* = 0.93) version of the Utrecht Work Engagement Scale for Students (UWES-S; Schaufeli et al., 2002). Students were asked to rate how often they had experienced the described feelings (e.g., “*I am enthusiastic about my studies.*”) on a 7-point Likert scale (from 0—*Never* to 6—*Every day*).

### 2.3. Procedures

The study was approved by the Ethics and Deontology Committee of the Faculty of Psychology, University of Lisbon (protocol code Ata No. 4/2024 approved on 23 January 2025). Participants were self-selected and provided written informed consent before data collection. Informed consent ensured data anonymity and confidentiality, and the possibility of withdrawal at any time during the study. To be eligible to participate, participants were required to be registered in a Portuguese higher education institution and to be aged between 18 and 35. Socio-demographic data were collected at the end of both the interviews and the survey. Participation was voluntary and anonymous, and data confidentiality was guaranteed. No compensation was offered.

Regarding qualitative data collection, the sampling process involved a combination of methods: in-person dissemination of the project by teachers to their classes; online dissemination of the study via email and social networks through the researchers’ direct networks; and a snowball sampling method through previous participants. Dissemination included an invitation letter outlining the study’s objectives and inviting students to participate. Those who expressed interest in participating were contacted by the second author to schedule the interview at a convenient time. Interviews were held in person and online (via videoconference). Data collection took place in the first semester of 2025. After authorization by the participants, the interviews were audio-recorded to facilitate transcription. The interviews lasted approximately 60 min. To determine the sample size, we followed a saturation-oriented approach. Over half the codes (85.7%) were identified in the first interview, and all codes included in the final (sub-)category system had emerged by the third interview, indicating an early stabilization of codes within this sample. This pattern should be interpreted as reflecting code-level recurrence rather than comprehensive thematic saturation. Audio recordings were assigned alphanumeric codes for each participant, and transcriptions were anonymized by removing any personal information that could identify participants. These procedures enabled contributions to be cross-referenced while ensuring data confidentiality and participants’ anonymity. Participants were informed about the destruction of recordings once the study was complete.

For the quantitative data collection, measures and socio-demographic questions were uploaded as an online survey using the Qualtrics platform (average response time: 15 min). The anonymous survey link, along with information regarding the study’s objectives, was distributed on social networks and launched via email to universities as well as departments, associations, and student unions, asking for their collaboration in the dissemination of the survey through mailing lists. To ensure online data quality and validity, we applied a data validation protocol controlling consistency of response and multiple response submissions, using text entry boxes to facilitate the detection of random answers, spam, or the use of autofill software, and establishing a minimum threshold of 5 min response completion time (Aust et al., 2013; Dewitt et al., 2018). A statement promoting honesty was included in the survey instructions, and a truthfulness item adapted from Cornell et al. (2012) (i.e., “*How many questions in this questionnaire did you answer truthfully?*”) was included at the end of the survey to mitigate social desirability bias and contribute to response validity screening (Larson, 2019). Response to the truthfulness item options ranged from 1—*Only some or none* to 5—*All of them*, with responses below or equal to 3 being considered unreliable. Responses that did not meet the data validation protocol criteria were deleted. The data collection process lasted approximately one month.

### 2.4. Data Analysis

For qualitative data analyses, given the exploratory and comprehensive nature of our research questions, we conducted a mixed deductive-inductive content analysis (Bardin, 2000) using NVivo 15. This approach enabled the systematic identification, organization, and interpretation of recurring categories and subcategories within participants’ responses. The corpus consisted of 16 *verbatim* transcriptions of the audio recordings. To enhance our understanding of the data and become familiar with its content, we conducted an active, progressive, in-depth reading of the corpus, identifying recording units (i.e., segments of meaningful content that were categorized, e.g., words, sentences, propositions). Initial and focused coding then defined the category and sub-category system. To ensure the analysis accuracy and internal validity, assumptions of exclusivity (each recording unit was assigned to only one (sub-)category), homogeneity (the category system was consistent and addressed the same topic), pertinence (the (sub-)categories answered the topic and research questions), objectivity (all (sub-) categories were operationally defined), and productivity (the category system yielded meaningful insights) were assured during the coding process (Bardin, 2000). Reliability was ascertained through inter-coder reliability and agreement (Campbell et al., 2013). An independent judge blindly coded a random selection of 20% of the recording units. Given the categorical nature of the category system, intercoder agreement was assessed using Cohen’s kappa. Results indicated substantial agreement between coders (*κ* = 0.73, 95% CI [0.62, 0.82]) (McHugh, 2012). Discrepancies were resolved through collaborative reflection until full agreement was attained. The use of a semi-structured interview script, independent coding procedures, and consensus meetings helped enhance analytic rigor and reduce the influence of individual researcher assumptions on data interpretation. Frequencies were used descriptively to indicate the relative prominence of categories and subcategories within this sample and should not be interpreted as indicators of statistical prevalence. Qualitative findings were subsequently integrated with quantitative results during interpretation to contextualize the mediation findings and provide a more comprehensive understanding of students’ adaptation and well-being.

Quantitative data analyses were performed using IBM SPSS Statistics (Version 29). Descriptive statistics (means, standard deviations) and preliminary analyses were employed to examine data distribution, identify missing values, and detect outliers. The *MCAR de Little* test revealed a completely random distribution of the missing values [*χ*^2^ (803) = 778.94, *p* = 0.730], which had little expression (<5%). Therefore, missing imputation did not occur, and a complete case analysis was performed (Tabachnick & Fidell, 2013). Regarding univariate normality and outliers, *Q-Q* plot analysis indicated a near-normal distribution of the data (i.e., |*z*| < 3; Kline, 2016). Internal consistency was assessed with coefficient omega (*ω*), with values ≥ 0.70 considered good (Crutzen & Peters, 2017). Pearson correlations were also computed to examine the relationships between variables. Correlations are considered small, moderate, and large for values around 0.10, 0.30, and 0.50, respectively (Cohen, 2013).

Given our sample size considerations and the complexity of the proposed theoretical model, we employed a generalized linear model (GLM) based mediation analysis using the jAMM module in *jamovi* (Version 2.3) rather than full structural equation modeling. This approach was selected for several reasons. First, preliminary power analysis indicated that testing the complete model with eight latent variables (five SEC, two self-care mediators, and one outcome variable) would require an impractically large sample size (*N* > 500) to achieve adequate statistical power and avoid identification issues (Wolf et al., 2013). Second, our primary research questions concerned the structural relationships between constructs rather than validation of the measurement model, making composite score analysis appropriate (Hayes, 2022). Composite scores were created by averaging items within each scale, a common practice when primary interest lies in structural relationships rather than measurement properties. Third, GLM mediation provides direct tests of specific indirect effects with bootstrap confidence intervals, which aligns with our hypotheses about mediation pathways (Preacher & Hayes, 2008). We employed bias-corrected bootstrapping with 5000 resamples to test the statistical significance of indirect effects (MacKinnon et al., 2004). This non-parametric approach provides more accurate confidence intervals than traditional normal theory approaches, particularly with smaller sample sizes (Preacher & Hayes, 2008). An indirect effect was considered statistically significant if the 95% confidence interval (CI) did not include zero. We report completely standardized effect sizes (*β*), facilitating comparisons across different predictors and pathways. Following Cohen’s (2013) guidelines, we interpret *β* values of 0.10, 0.30, and 0.50 as representing small, medium, and large effect sizes, respectively. Analyses were conducted with a significance level of *p* < 0.05.

## 3. Results

### 3.1. Stage 1—Qualitative Data Analysis

#### Higher Education Students’ Academic Adaptation

**Figure 2:**
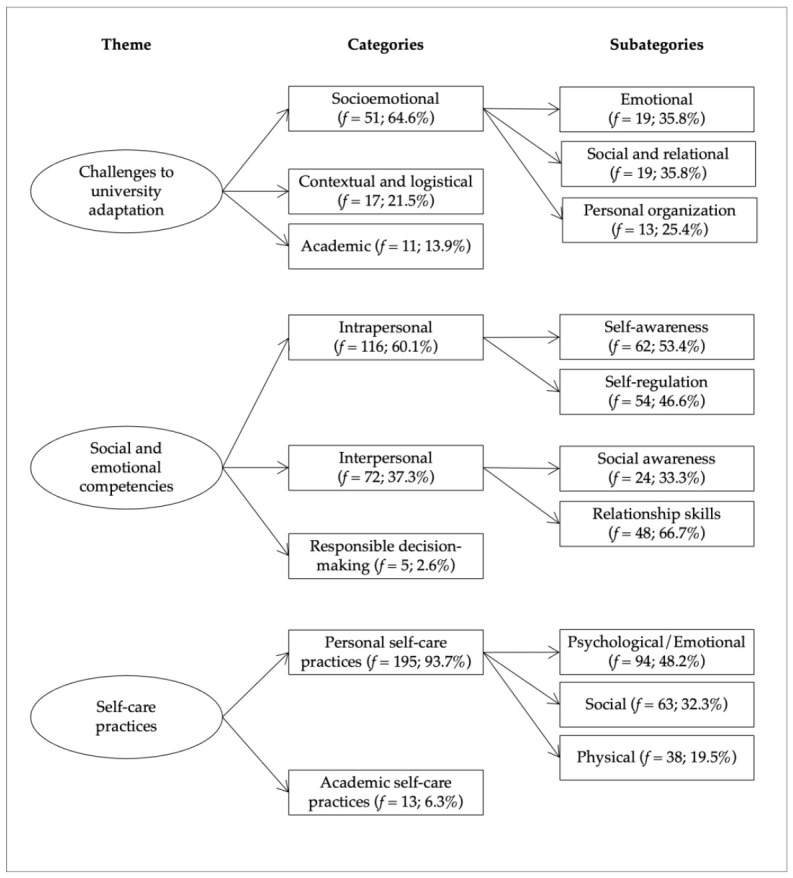
Data structure (Total recording units = 480, 100%).

Content analysis yielded a framework of interrelated themes that captures the experiences of students transitioning to higher education. This encompasses the specific challenges they encounter in their daily lives and the skills and strategies they deem essential to overcome these challenges and foster their well-being. The analysis produced 8 categories and 10 subcategories, organized into three higher-order categories: Challenges to university adaptation, Social and emotional competencies, and Self-care practices. A total of 480 recording units were coded. The data structure is depicted in Figure 2, and an extended summary of illustrative interview quotes for each (sub-)category is available in Table S1 in the Supplementary Materials. Below, we present the results, which were organized to address our research questions.

*RQ1:* What are the main challenges perceived by higher education students in adapting to university?

Regarding *Challenges to university adaptation*, the analysis revealed that the main challenges experienced by participants were primarily socioemotional (64.6%). Specifically, 75.0% of the higher education students reported *Emotional* difficulties (35.8%), describing negative emotional experiences, as well as difficulties in regulating their emotions, managing expectations, and mobilizing appropriate coping strategies (e.g., *“These adaptations have been very difficult for me to cope with; I start to doubt whether I’ll be able to get through this, thinking I don’t have the ability, that I lack the skills, that it’s going to be hard*”, P08). These were followed by *Social and relational* challenges (35.8%), referred to by 68.8% of the participants. At this level, students described experiencing difficulties in initiating, integrating, and maintaining social relationships with peers and managing prior relationships after attending university (e.g., “*It still took me a little while to find people I could relate to*”, P10). The difficulty of forming new relationships while distancing from previous ones may contribute to higher education students’ social vulnerability, as the literature identifies social integration as an important factor influencing well-being and the decision to remain in university (e.g., Purnamasari et al., 2022). The absence of a social support network has also been identified as a factor that contributes to vulnerability to stress, as well as feelings of loneliness and uncertainty (Campos et al., 2024; Farias & Almeida, 2020).

Adding to these, students also described challenges related to *personal organization* (25.4%), *contextual and logistical* changes (21.2%), and *academic* hurdles (13.9%). These findings highlight that transitioning to university demands a qualitative leap in executive skills, such as time management, autonomy, and planning, as a lack of these skills can lead to academic stress and difficulties in balancing personal life and studies (Purnamasari et al., 2022). In conclusion, our findings suggested that higher education students were not only adapting to a new environment but also navigating a critical phase of psychosocial development (Erikson, 1982). Whether this was due to the need to develop skills, consolidate their identity, or build deep bonds with others, higher education students were facing one of the most demanding phases of their lives.

*RQ2:* Which social and emotional competencies do higher education students perceive as most relevant to promoting their personal and academic well-being?

Following the SEC model proposed by Durlak et al. (2015, 2025), the theme concerning higher education students’ *Social and emotional competencies* was organized into three main categories: Intrapersonal (60.1%), Interpersonal (37.3%), and Responsible Decision-Making (2.6%). *Intrapersonal* skills were the most discussed topic among the participants, comprising *Self-awareness* (53.4%) and *Self-regulation* (46.6%). *Self-awareness* was the most frequently mentioned SEC, with a predominant focus on accurate self-perception (e.g., “*I’ve learned a lot, I’ve grown a lot, and I’ve gotten to know myself quite well”*, P12), along with descriptions of emotional awareness (“*Anxiety really wears me down; I’m an anxious person*”, P09) and optimism (“*We have to always look ahead and see the light at the end of the tunnel, even if the tunnel is lon*g”, P01). As for *Self-regulation*, students mainly mentioned the mobilization of organizational skills (e.g., “*I also try to always write down everything I have to do …*”, P04) and strategies related to emotional and behavioral regulation (e.g., “*I need to convince myself that I’m not in a very time-sensitive situation, otherwise I’ll get stressed* …”, P06).

Concerning *Interpersonal* skills, references to *Relationship skills* were particularly prevalent (66.7%), particularly in relation to establishing and maintaining relationships (“*I try to stay involved in people’s lives, even though I can’t be with them every day. For example, I often call my famil*y”, P05), and managing conflict (e.g., “(…) *I try very hard to welcome the other person* (…)”, P15). Although less frequent, *Social awareness* skills were also described, particularly in relation to recognizing available resources and support (e.g., “*I know I have a good network of support and assistance*”, P13), understanding emotions in others (e.g., “*I can tell when people are feeling a little down, just by the way they look*”, P06) and perspective-taking (e.g., “*I always try to understand the other side*”, P09) as sources of well-being. *Responsible decision-making* emerged as the SEC least frequently mentioned by the participants (2.6%), with only 25.0% of students referencing it.

The emphasis placed on intrapersonal skills compared to interpersonal skills and to responsible decision-making aligns with the idea that transitioning to higher education shifts the focus from external regulation to an increased need for internal regulation (Conley & Donahue-Keegan, 2025). As Conley and Donahue-Keegan (2025) argue, the university environment requires students to continually use self-awareness and self-regulation skills to navigate a less structured and more demanding context. The strong internal significance of self-awareness suggests that, for participants, realistic recognition of one’s own capabilities, limits, and internal states was seen as a precondition for their well-being, in line with the literature, which defines self-awareness as the foundation for effective self-regulation (e.g., Elmi, 2020). Regarding self-regulation, the emphasis on emotional and behavioral regulation, as well as organizational skills, suggests that participants linked their well-being to emotional and behavioral control and practical strategies for managing studies, time, and tasks. This is consistent with recommendations for interventions in higher education that combine emotional regulation training with the development of organizational habits and active participation (Socas, 2017; Wyatt & Bloemker, 2013).

Although less prominent, the specific interpersonal skills highlighted by participants suggest that they prioritize understanding and interpreting social and institutional dynamics to better adapt to their operating contexts. Recognizing emotions in others and understanding different perspectives can serve as tools for social navigation. These skills enable students to adapt to a socially diverse and complex environment, collaborate effectively on group tasks, activate support networks, approach others appropriately, co-create a psychologically safe learning environment, and build a sense of community, which are fundamental to their well-being (Elmi, 2020; Matthewman-Isgrigg, 2024; Socas, 2017; Wyatt & Bloemker, 2013).

*Responsible decision-making* appears to have been considered less relevant by participants than other social and emotional competencies when considered as a standalone domain. This may reflect participants’ tendency to view appraisal, reflection, and problem-solving as part of intrapersonal or interpersonal competencies. This finding highlights the difficulty of strictly compartmentalizing the five areas of SEC, given that they are often engaged simultaneously.

*RQ3:* Which self-care practices are considered most important by higher education students to promote their personal and academic well-being?

Regarding the last theme, *Self-care practices* were split into *Personal self-care* (93.7%) and *Academic self-care* (6.3%). The former describes practices used by participants to promote their personal well-being (e.g., “*Getting involved in activities I enjoy doing*”, P14), while the latter includes references to practices that promote academic well-being (e.g., *“I am currently taking the victim support technician course at APAV*”, P09). Within *Personal self-care*, participants mainly described *Psychological/Emotional* self-care practices, such as engaging in self-development activities, leisure, emotional management, and restorative breaks (48.2%; e.g., “*Sometimes I start a TV show* (…), *or I go to bed, so it’s that moment to unwind before actually resting*”, P02). Additionally, references were also made to *Social* self-care practices, including seeking social interaction, relational rituals, intimacy, and affective validation (32.3%; e.g., “*I’m learning French right now. My boyfriend lives in France. So that’s also … it’s a moment for the two of us* …”, P09). Although less frequent, there were also references to *Physical* self-care practices, including caring for one’s nutrition, sleep, body care, and physical activity (19.5%; e.g., “*I eat a healthy, varied diet*”, P14).

The results showed that higher education students recognized and engaged more frequently in personal self-care practices than academic self-care practices. This suggests that participants conceived of self-care as predominantly personal and intimate actions, rather than as actions directly linked to the academic context. This finding is consistent with studies documenting the tendency to neglect professional and academic self-care practices (Lee et al., 2020) and highlights the importance of intervention in this area.

Within personal self-care, practices of a psychological and emotional nature were favored, suggesting that these students prioritize promoting mental and emotional balance in their self-care. However, it is important to note that this finding may have been influenced by the fact that participants were psychology students who may have found it easier to name and report on psychological and emotional self-care practices. It may also have been influenced by the growing recognition of mental health issues in a university context (Roxas, 2023; Zhong & Xie, 2023). Further investigation is therefore needed.

Despite the primacy of the psychological/emotional domain, the expression of social and physical self-care practices suggests that participants recognized their interdependence in promoting their personal well-being, an idea already theorized by Lee and Miller (2013). In particular, the importance of social self-care is widely supported by literature, which identifies it as an essential dimension for students’ well-being (e.g., Tram et al., 2025). By contrast, the scarce mention of academic self-care in the participants’ discourse suggests that they may not have recognized academic practices as a form of self-care. This discrepancy may be due, in part, to a difference between the operational definitions of self-care and how students subjectively understand it.

Overall, the qualitative findings provided an initial understanding of the challenges experienced by higher education students and the social and emotional competencies and self-care practices they perceived as most relevant for their well-being. Building on these insights, the quantitative phase examined the relationships between SEC, self-care practices, and personal and academic well-being in a larger sample of students.

### 3.2. Stage 2—Quantitative Data Analyses

#### 3.2.1. Relation Between Students’ Social and Emotional Competencies, Self-Care Practices, and Personal and Academic Well-Being: Correlation Analysis

Descriptive statistics (Table 1) indicate moderate to moderate-high levels of SEC, with self-regulation (*M* = 6.36) and positive relationship skills (*M* = 6.93) presenting slightly lower levels. Both personal and academic self-care practices showed moderate levels (around the scale’s midpoint), with lower scores for academic self-care (*M* = 3.35). Regarding students’ well-being, personal well-being scores were marginally higher (*M* = 2.58) than the midpoint of the scale (2.5), and academic well-being scores were moderately high (*M* = 3.56, midpoint = 3).

Inter-correlations between students’ social and emotional competencies, self-care practices, and personal and academic well-being were generally positive and moderate to large (Table 1). Strongest associations were found between self-regulation and academic self-care (*r* = 0.51), personal (*r* = 0.64) and academic well-being (*r* = 0.61); between personal self-care and personal well-being (*r* = 0.57); and between academic self-care and personal (*r* = 0.61) and academic well-being (*r* = 0.50). Importantly, although SEC dimensions and self-care indicators were positively associated, the magnitude of these correlations (*r* = 0.24–0.51) suggests that these constructs share variance, as theoretically expected, while remaining conceptually and empirically distinct. Multicollinearity diagnostics further supported this interpretation, with variance inflation factor (VIF) values ranging from 1.61 to 2.57, remaining below commonly accepted thresholds (VIF < 3; Menard, 2002). This suggests that, despite their association, these variables do not reflect redundant constructs.

#### 3.2.2. Test of Proposed Conceptual Model: Mediation Analysis

##### Personal Well-Being

The hypothesized parallel multiple mediation model explained substantial variance in personal well-being (*R*^2^ = 0.42), indicating a robust predictive relationship. Both self-care mediators contributed significantly to personal well-being: personal self-care (*β* = 0.227, *p* = 0.001) and academic self-care (*β* = 0.273, *p* < 0.001) (Table 2).

Three key findings emerged from this mediation analysis. First, self-regulation exhibited the strongest association with personal well-being (total *β* = 0.678, *p* < 0.001), demonstrating both a substantial direct effect (*β* = 0.479, *p* < 0.001) and significant indirect effects through both academic self-care (*β* = 0.124, *p* = 0.008) and personal self-care (*β* = 0.076, *p* = 0.029) (Table 3). Second, positive relationship skills showed an indirect-only mediation pattern, significantly influencing personal well-being through academic self-care (*β* = 0.092, *p* = 0.022) but not through personal self-care or directly (Table 3). Third, for personal well-being, neither self-awareness, conflict management, nor responsible decision-making showed statistically significant indirect, direct, or total effects within the multivariate mediation model, as all corresponding 95% confidence intervals encompassed zero (Table 3). This pattern should be interpreted considering the positive bivariate associations observed for some variables (Table 1), suggesting that their effects may be accounted for by shared variance with other competencies included in the model. Notably, despite a positive bivariate correlation between self-awareness and personal well-being being registered (*r* = 0.27; Table 1), a negative (though non-significant) direct (*β* = −0.112, *p* = 0.083) and total (*β* = −0.141, *p* = 0.105) effects emerged in the mediation model. This pattern may reflect a suppression effect or shared variance with other SEC dimensions, particularly with self-regulation. Age and academic year were included as covariates in the model. Neither variable showed significant indirect, direct, or total effects on students’ personal well-being.

##### Academic Well-Being

The hypothesized parallel multiple mediation model accounted for a substantial proportion of the variance in academic well-being (*R*^2^ = 0.46), suggesting a strong predictive relationship. Of the two proposed mediators, only academic self-care emerged as a significant predictor of academic well-being (*β* = 0.208, *p* = 0.004), whereas personal self-care did not demonstrate a significant direct effect (*β* = 0.108, *p* = 0.109) (Table 4).

Self-regulation exhibited the strongest total effect on academic well-being (*β* = 0.692, *p* < 0.001), suggesting that higher levels of self-regulation are associated with increased academic well-being. This effect comprised a substantial direct pathway (*β* = 0.561, *p* < 0.001) as well as a significant indirect effect through academic self-care (*β* = 0.094, *p* = 0.011), supporting a partial mediation process (Table 5). Similarly to the findings for personal well-being (cf. Table 3), positive relationship skills showed an indirect-only mediation pattern, significantly influencing academic well-being through academic self-care (*β* = 0.073, *p* = 0.020) but not through personal self-care or directly. Self-awareness, however, exhibited a negative direct (*β* = −0.198, *p* = 0.009) and total (*β* = −0.222, *p* = 0.005) effects on academic well-being. The indirect effects of self-awareness on academic well-being through self-care practices were non-significant.

The multiple mediation model for academic well-being indicated that personal self-care did not operate as a significant mediator for any of the social and emotional skills examined, as all indirect effects through personal self-care included zero within their 95% confidence intervals (Table 5). Moreover, conflict management and responsible decision-making showed no significant indirect, direct, or total effects. Age and academic year were included as covariates in the model. Neither variable showed significant indirect, direct, or total effects on students’ academic well-being.

## 4. Discussion

### 4.1. Interpretation of Key Findings

This study was based on the understanding that the transition to higher education represents a period during which academic demands overlap with key psychosocial tasks of emerging adulthood (Campos et al., 2024; Casanova et al., 2020; Erikson, 1982), making higher education students more vulnerable to mental health problems (e.g., Duffy et al., 2020; Farias & Almeida, 2020; Fonte & Macedo, 2021). Thus, it is important to understand how students’ well-being—defined as a dynamic process of multidimensional adjustment (Purnamasari et al., 2022)—can be promoted and sustained. In this context, a growing body of research has highlighted the importance of developing social and emotional competencies in the university setting (e.g., Conley & Donahue-Keegan, 2025; Elmi, 2020). The mobilization of these skills may facilitate engagement in self-care practices, which are considered intentional and proactive processes for promoting well-being (Lee & Miller, 2013; Markin, 2022). However, an empirical gap remains regarding the interplay between social and emotional competencies, self-care practices, and higher education students’ well-being. Thus, through a mixed-methods study, we sought to address this gap by exploring which SEC and self-care practices, from these students’ perspective, contribute most to their well-being. Moreover, building on prior literature and informed by the insights generated in the qualitative phase, we investigated the mediating role of self-care practices in the relationship between students’ SEC and their well-being.

#### 4.1.1. Higher Education Students’ Well-Being: Integrated Findings on Challenges, Social and Emotional Competencies, and Self-Care Practices

The integration of qualitative and quantitative findings yielded a comprehensive view of higher education students’ well-being, highlighting how social and emotional competencies and self-care practices were mobilized by students to face perceived challenges and promote their personal and academic well-being. Firstly, regarding RQ1 (i.e., *What are the main challenges perceived by higher education students in adapting to university?*), both strands of data consistently portray the transition to higher education as a demanding and potentially distressing period, particularly at a socioemotional level. Qualitative findings emphasized emotional and relational difficulties, and quantitative results corroborated this pattern, suggesting only moderate levels of personal and academic well-being. These findings are consistent with previous research documenting significant levels of stress, academic burnout, and mental health problems among university students (e.g., Gebregergis & Csukonyi, 2025; Ribeiro et al., 2019). The alignment between perceived challenges and well-being indicators reinforces the interpretation of university adaptation as a critical, risk-laden developmental phase. This interpretation is also consistent with transactional models of stress and coping (Lazarus & Folkman, 1984), which propose that psychological adjustment depends on how individuals appraise demands and mobilize personal resources to manage them. Nevertheless, these results warrant further investigation, as the stronger emphasis on socioemotional challenges may reflect what students find easier to recognize and describe. This is of particular relevance considering that our qualitative findings stemmed from a sample of exclusively psychology students. Also, emotional experiences are often more salient and socially validated, whereas academic or structural challenges may be considered part of standard university life and therefore less frequently mentioned.

Regarding RQ2 (i.e., *Which social and emotional competencies do higher education students perceive as most relevant to promoting their personal and academic well-being?*), the qualitative data revealed that intrapersonal competencies, particularly self-awareness and self-regulation, were described by higher education students as the most significant skills for promoting their well-being. In the interpersonal domain, relationship skills were held in the highest regard. Simultaneously, the quantitative results suggested that students self-reported lower levels of self-regulation and positive relationship skills. This apparent discrepancy may stem from the fact that the competencies students considered most relevant to their well-being were, to some extent, those in which they felt least proficient. Furthermore, mediation analyses indicated that self-regulation and positive relationship skills were significant predictors of personal and academic well-being. This aligns with prior evidence (e.g., Oliveira et al., 2025) and reinforces the explanatory relevance of these skills. The convergence across methods is noteworthy, as the qualitative findings highlighted the importance of intrapersonal competencies and relationship skills for students’ well-being, while the quantitative analyses identified self-regulation and positive relationship skills as particularly relevant predictors of personal and academic well-being. These findings emphasize the importance of institutionally coordinated intervention initiatives within higher education contexts to explicitly promote these competencies and support students’ needs.

Regarding RQ3 (i.e., *Which self-care practices are considered most important by higher education students to promote their personal and academic well-being?*), both qualitative and quantitative findings show that students reported more personal than academic self-care practices. The convergence of findings across methods strengthens the interpretation that self-care practices are predominantly understood and enacted by students within the personal domain rather than the academic domain. Qualitative data showed a clear predominance of psychological and emotional self-care practices, a pattern mirrored quantitatively by moderate overall self-care levels and comparatively lower involvement in academic self-care. The literature offers plausible explanations for this distribution. Psychological and emotional self-care practices (e.g., breathing exercises or journaling) tend to be self-contained, flexible, low-cost, and easily integrated into demanding schedules, thereby facilitating adherence (Bishop et al., 2024). Structural barriers, including time constraints and financial limitations, could further reinforce the preference for accessible and individually manageable strategies (Bishop et al., 2024). At the same time, this emphasis may also reflect an adaptive response to high emotional demands, consistent with the described challenges. In this sense, self-care practices emerge not merely as a lifestyle preference but as a regulatory response to experienced distress. Nonetheless, alternative interpretations should also be considered. The emphasis on psychological self-care may be influenced by sample characteristics, as psychology students may be more likely to identify and report these practices. Furthermore, other forms of self-care practices, such as physical or academic practices, may be mentioned less frequently, not because involvement in these practices is lower, but because they are not clearly identified as ‘self-care’.

Moreover, the limited reference to academic self-care practices requires critical interpretation. Rather than reflecting individual preferences alone, this finding may suggest a structural gap in the way self-care is understood and implemented within higher education. The existing literature argues that promoting academic self-care may benefit from explicit integration into the curriculum and from the active involvement of faculty members in modelling and supporting these practices to effectively promote students’ well-being (Brown & Archbell, 2025). High levels of student stress, academic burnout, and mental health problems (e.g., Gebregergis & Csukonyi, 2025; Ribeiro et al., 2019) also suggest that individual effort alone is insufficient without systemic support. From this perspective, the reduced expression of academic self-care in this study may signal both limited student involvement and a deficit in the institutional provision, visibility, or legitimization of such practices.

Finally, qualitative descriptions of students’ involvement in self-care practices suggest a reactive approach, with self-care being used in response to stress rather than as part of a regular preventive routine. This reactive pattern may limit the long-term effectiveness of self-care, potentially leading to cyclical experiences of overload and recovery. Taken together, the findings suggest the need for multi-level intervention strategies. While students demonstrate awareness of, and involvement with, certain adaptive practices, particularly at the intrapersonal and psychological levels, there is clearly a need for institutional investment in the structured promotion of social and emotional competencies and self-care practices, especially within the academic domain. This highlights the need for interventions that move beyond an exclusive focus on individual responsibility, incorporating systemic institutional efforts to normalize, structure, and actively promote social and emotional learning and self-care practices. This would foster a more integrated and sustainable model of student well-being.

#### 4.1.2. The Interplay Between Students’ SEC and Self-Care Practices to Enhance Their Personal and Academic Well-Being

Building on the qualitative findings, which highlighted the perceived importance of intrapersonal competencies, relationship skills, and self-care practices for students’ adaptation and well-being, the mediation analyses provided further insight into the mechanisms linking these variables. Mediation analysis results suggest that different social and emotional competencies operate through distinct pathways to influence higher education students’ personal well-being. Self-regulation emerged as the most robust predictor, exerting a strong total effect on personal well-being by enhancing it both directly and by promoting both academic and personal self-care practices. From a coping perspective (Lazarus & Folkman, 1984), self-regulation may represent an important personal resource that enables students to manage academic and socioemotional demands more effectively, partly through engagement in adaptive self-care practices. These findings indicate a pattern of partial mediation, in which self-regulation retains a direct association with personal well-being while simultaneously operating through self-care practices. In contrast, positive relationship skills contribute to personal well-being primarily by facilitating academic self-care, as only this pathway reached statistical significance. This suggests a more domain-specific indirect effect, in which interpersonal competencies appear to enhance personal well-being specifically by promoting effective academic self-care practices (e.g., organization, help-seeking, and engagement with academic responsibilities).

An unexpected finding was the negative (though non-significant) association between self-awareness and personal well-being. While this pattern may reflect statistical suppression, particularly given the positive bivariate correlation between these variables, it may also indicate that, after accounting for shared variance with other SEC (e.g., self-regulation), self-awareness did not make a unique contribution to the prediction of personal well-being. Moreover, the possibility that certain aspects of self-awareness (e.g., hyper-self-focus) could potentially undermine well-being when other social and emotional competencies are controlled cannot be ruled out. In fact, similar results are found in prior literature (Oliveira et al., 2025; Simsek, 2014). It seems that specific dimensions of self-awareness (e.g., self-reflection), especially if not accompanied by effective self-regulatory strategies, could be associated with increased self-criticism or rumination, hindering higher education students’ well-being (Simsek, 2014). Although this finding warrants further theoretical and empirical investigation, it underscores the importance of distinguishing between adaptive reflective awareness and maladaptive self-focused attention.

Regarding academic well-being, results yielded a partially convergent yet conceptually distinct pattern. Both self-regulation and positive relationship skills appear to exert part of their influence on academic well-being by fostering academically oriented self-care practices, thereby reinforcing the functional relevance of this mediator in the academic domain. In this model, academic self-care emerged as the active explanatory mechanism, clarifying how higher levels of self-regulation and positive relationship skills translate into students’ higher academic well-being. These findings highlight that self-care practices, such as structured study routines, proactive help-seeking, and sustained involvement in academic tasks, constitute the proximal behavioral pathways through which SEC contribute to students’ academic well-being.

Consistent with the findings observed for personal well-being, self-regulation remained the most robust predictor of students’ academic well-being. Both its direct and indirect effects through academic self-care were statistically significant, indicating partial mediation. This pattern suggests that self-regulation enhances academic well-being not only by promoting students’ involvement in academic self-care but also through additional mechanisms, potentially including improved emotional and behavioral regulation, goal setting and achieving, adaptability, and organizational skills (Oliveira et al., 2023). The dual pathway underscores the centrality of self-regulatory competencies in higher education students’ academic adjustment.

Interestingly, within the present model, personal self-care practices were found to lack explanatory power in predicting academic well-being. Neither direct nor indirect effects through personal self-care practices reached statistical significance. The absence of effects through this pathway suggests that, in our model, personal self-care practices do not constitute a meaningful mechanism for explaining variance in students’ academic well-being. This finding reinforces the domain-specific nature of the mediation process, whereby academically oriented behaviors, rather than general self-care practices, account for the translation of social and emotional competencies into academic well-being.

Following the pattern observed in the personal well-being model, a negative direct association between self-awareness and academic well-being was found. Although self-awareness was positively correlated with academic well-being at the bivariate level, its negative regression coefficient in the full model suggests that, when controlling for the remaining SEC and mediators, higher levels of self-awareness were associated with lower academic well-being, suggesting a potential suppression effect or complex interplay among predictors that warrants further theoretical and empirical examination. Although the observed effect could reflect a statistical artefact, the observed tendency suggests a pattern (e.g., Oliveira et al., 2025; Simsek, 2014). Further theoretical development and empirical investigation of potential substantive processes within the broader SEC framework are required.

Taken together, and answering RQ4 (i.e., *Do personal and academic self-care practices mediate the relationship between students’ SEC and their personal and academic well-being?*), our findings suggest that the mediating role of self-care practices is domain-specific. While both personal and academic self-care practices emerged as significant mediators of the relationship between SEC and personal well-being, for academic well-being, mediation occurred exclusively through academic self-care practices. In conclusion, our findings suggest that self-care practices act as one important behavioral mechanism linking SEC to well-being; however, their impact depends on the type of well-being and social and emotional competencies under consideration. These results, adding to the observed moderate mean levels of SEC and self-care practices, underscore the need for tailored interventions that bolster students’ self-regulation, positive relationship skills, and self-care practices, while also stressing the need to further examine when self-awareness may be associated with less adaptive outcomes.

### 4.2. Limitations

Despite its contributions, our study has several limitations. First, convenience sampling was used in both phases. The qualitative study relied on a small and relatively homogeneous sample of psychology students, predominantly young, female, Portuguese, and postgraduate students. Although this approach facilitated efficient data collection, it may have increased selection bias and limited representativeness and generalizability. Moreover, the predominance of psychology students and female participants may have influenced the content of responses, as these groups may be more familiar with or more likely to articulate concepts related to emotions, well-being, and self-care. Given the small and relatively homogeneous qualitative sample, thematic development may also have been constrained, and the findings should be interpreted as exploratory and context-bound. To partially address this limitation, quantitative data were collected from students across different universities and degree programs throughout the country. Nevertheless, although diverse, the quantitative sample retained some imbalance in its composition, with a predominance of female and HASS students enrolled, which may have had an impact on the observed associations between SEC, self-care practices, and well-being. It should also be acknowledged that some important variables that may influence well-being and self-care practices were not controlled in the mediation model. Although age and academic year were included as covariates, other potentially relevant factors (e.g., gender, socioeconomic background, living arrangements, field of study, and previous mental health history) were not considered and may have influenced the observed associations. Contextual and institutional factors (e.g., academic workload, institutional support, teaching practices) were also not explored in this study. Therefore, future studies should continue to explore the relationship between students’ SEC, self-care practices, and well-being across different samples, while accounting for additional factors that may impact these relationships.

Second, despite our efforts to enhance response validity, reliance on self-report measures may have increased the risk of recall and social desirability biases. Students may have emphasized experiences, skills, and practices that are more salient, socially accepted, or easier to articulate, while underreporting academic or structural challenges. Although the mixed-methods design strengthened the study by integrating qualitative and quantitative evidence, the two strands involved independent samples. Converging findings suggest consistency, but alternative explanations cannot be entirely ruled out, as some patterns may reflect differences in how constructs are understood or measured.

Third, the cross-sectional design limits causal inference. Longitudinal studies are needed to clarify the directionality of the relationships among SEC, self-care, and well-being. Additionally, the use of composite scores may have reduced sensitivity to item-level variability, latent measurement error, and potential multidimensionality within the assessed constructs. Future studies could employ latent variable approaches to further examine these relationships. Relatedly, the personal self-care subscale showed comparatively lower internal consistency (*ω* = 0.67). Although this value falls below the conventional 0.70 criterion, it exceeds the minimum threshold of 0.60 often considered acceptable in exploratory research (Crutzen & Peters, 2017). The scale was therefore retained due to its theoretical relevance, acceptable reliability for exploratory research, and adequate overall performance. Nevertheless, lower reliability may have attenuated observed associations and reduced the precision of mediation estimates, warranting caution when interpreting findings involving this dimension.

Finally, despite the implementation of procedures to ensure coding validity and reliability, qualitative interpretation remains inherently subjective. Although a semi-structured interview script, independent coding procedures, and consensus meetings were used to enhance analytic rigor, interviewer and researcher perspectives may nevertheless have influenced data collection, coding, and interpretation. The use of frequency analysis may also have overstated the prevalence of some viewpoints. Moreover, the operationalization of SEC and self-care may not fully align with students’ subjective understandings of these constructs, potentially leading to non-recognition or underreporting of certain behaviors. For example, the relatively limited reference to responsible decision-making compared with intrapersonal and interpersonal competencies suggests that students may perceive these domains as overlapping rather than distinct. This challenge was also evident during data analysis. Although Cohen’s kappa indicated substantial agreement, discrepancies identified before the consensus meeting highlighted difficulties in distinguishing between competencies related to behavior management (particularly self-regulation) and self-care practices aimed at psychological and emotional management. This calls for reflection on how these competencies are operationalized, assessed, and taught in higher education settings. More broadly, some SEC dimensions, particularly self-regulation, may share conceptual features with certain self-care domains, including academic self-care, raising questions about their discriminant validity. Furthermore, while SEC and self-care were conceptualized as distinct constructs and generally showed moderate associations, the strongest correlation observed between self-regulation and academic self-care was large according to conventional benchmarks (*r* = 0.51), suggesting conceptual relatedness without indicating construct redundancy. Consequently, some shared variance is theoretically expected, and the observed mediation effects should be interpreted with consideration of the potential conceptual overlap between these constructs. Although multicollinearity diagnostics indicated acceptable levels of collinearity (VIF < 3), supporting the interpretation of non-redundancy, these findings should still be interpreted considering the conceptual proximity between these constructs. Future research should continue to examine their discriminant validity and clarify the mechanisms by which specific social and emotional competencies contribute to self-care behaviors and well-being outcomes.

### 4.3. Study Impact

Our study makes important contributions to research and practice by supporting the idea that social and emotional competencies and self-care practices can promote and sustain the personal and academic well-being of higher education students.

From a theoretical perspective, our study builds on existing literature on the transition to university by integrating social and emotional competencies, self-care practices, and well-being within a single framework based on students’ lived experiences. This addresses a gap in the literature, particularly in the Portuguese context, where these dimensions are often examined separately. Our results support a more systemic view of student adjustment, suggesting that student well-being is best understood as a combination of interrelated elements. Challenges, social and emotional competencies, and self-care practices appear to influence each other dynamically. For example, lower levels of self-regulation can make individuals more vulnerable to stress, leading them to rely more heavily on self-care strategies. This implies that future models should adopt a more integrated approach that considers these interactions rather than treating each component separately. The findings also provide further insight into the nature of the transition to higher education. The prominence of social, relational, and organizational challenges is consistent with developmental models that emphasize identity formation and the search for intimacy, while also supporting the idea that this transition involves a shift toward greater internal regulation. In this sense, adjusting to university involves meeting both academic and developmental demands.

Additionally, the findings suggest that self-care practices can be understood as a behavioral pathway through which social and emotional competencies may be expressed in everyday life, while remaining analytically distinguishable from them. In this sense, self-care appears to operate as a mechanism through which SEC such as self-awareness and self-regulation are translated into actions that support well-being. The moderate correlations observed between self-care and both self-awareness and self-regulation, and acceptable levels of multicollinearity, reinforce this interpretation. While analytically distinguishable, these constructs are conceptually intertwined, suggesting that students who are more attuned to their internal states and better able to regulate their behavior are also more likely to engage in adaptive self-care practices. This perspective supports the role of self-care as a mediating mechanism linking SEC to well-being outcomes, suggesting that the impact of these competencies may be, at least partly, enacted through everyday regulatory behaviors. At the same time, these findings support a multidimensional conceptualization of students’ well-being that extends beyond academic performance to incorporate emotional and relational functioning as central components. Furthermore, relatively low involvement in self-care practices may reflect contextual and individual barriers, such as time constraints or situational demands. This highlights the need for further research to better understand the factors that may be limiting students’ ability to engage in self-care and support their well-being.

From a practical standpoint, the results underscore the need to position student well-being as a core institutional priority, namely due to its association with academic success (e.g., Marques-Pinto et al., 2025; Schaufeli et al., 2002). This supports investment in coordinated strategies that promote social and emotional competencies and foster supportive learning environments. One of the most relevant insights is the clear gap between what students identify as important and their reported level of functioning. The discrepancy between the importance attributed to certain competencies (e.g., self-regulation and relationship skills) and the levels of proficiency reported by students suggests a possible gap between awareness and effective implementation. Students seem to recognize what is important for their well-being, but they may not yet have fully developed the ability to apply these competencies consistently. These results suggest that institutional efforts should focus on targeted and structured interventions to develop these competencies. Such interventions may be more effective if they combine emotional regulation with practical skills, such as time management, planning, and navigating social dynamics within the university context. Importantly, embedding the development of these competencies into the curriculum, especially in the early stages of higher education, could increase their effectiveness. This may be achieved not only through structured initiatives and targeted programs, but also through the modelling and reinforcement of social and emotional competencies by instructors in everyday teaching and learning interactions, thereby strengthening personal and interpersonal resources that support students’ adaptation and success in higher education (Durlak et al., 2025). These efforts should, nevertheless, be situated within a broader systemic approach to student well-being, recognizing that students’ experiences are shaped by multiple interacting contexts (e.g., home/family).

The findings also highlight the need to reconsider how self-care is approached in higher education. Although students report engaging in personal self-care practices, particularly psychological and emotional practices, academic self-care appears to be less visible and adopted less frequently. This does not necessarily mean that students are neglecting it entirely, but rather that these practices may not be clearly defined or recognized as forms of self-care. Therefore, there is a case for making academic self-care more explicit within teaching and learning processes. This could involve helping students to develop sustainable study routines, manage their workload more effectively, and recognize the direct link between these behaviors and their well-being. In parallel, faculty members may play an important role by modelling realistic expectations, promoting balanced work habits, and normalizing difficulties associated with academic adjustment. The fact that most self-care practices were described as a reactive response to stress rather than as part of a consistent preventive approach also highlights the importance of preventive intervention. Overall, these findings suggest that promoting student well-being requires a more balanced approach combining individual skill development with stronger institutional support and refining theoretical models to better reflect how students experience and engage with these processes.

## 5. Conclusions

In a two-phase mixed-methods study, we employed an exploratory sequential design with the intent of providing a deeper understanding of the interplay of higher education students’ social and emotional competencies, self-care practices, and personal and academic well-being. The main findings revealed that students perceived entering higher education as less of an academic challenge and more of a transitional phase defined by socioemotional, relational, and personal organizational challenges. These findings are consistent with literature documenting the importance of social integration as a critical factor for well-being and retention in higher education (e.g., Purnamasari et al., 2022). Similarly, difficulty in balancing academic and personal commitments reflects the need for self-regulation skills. The absence of these skills has been identified as a robust predictor of academic stress (Fonte & Macedo, 2021; Purnamasari et al., 2022). This is reinforced by mediation analysis, which showed that self-regulation had the strongest direct and indirect effects on predicting students’ personal and academic well-being, while positive relationship skills primarily operated through academic self-care. However, while the interviewed students particularly valued intrapersonal skills, specifically self-awareness, as essential to their well-being, the quantitative results suggested an inverse correlation between self-awareness and well-being. These results point to the possibility of an indirect effect of self-awareness on well-being through self-regulation skills and emphasize the importance of explicitly teaching self-regulation skills in the context of interventions in higher education (Conley & Donahue-Keegan, 2025).

An analysis of student-reported self-care practices revealed that those most valued for promoting well-being were of a personal nature, focusing on psychological and emotional balance. This can be explained as an adaptive and pragmatic response to a highly stressful environment. However, this may also be the result of barriers to other self-care practices, particularly social practices and physical exercise, as well as a reflection of how university students conceptualize self-care. Therefore, further studies must be conducted to identify potential barriers to and enhancers of self-care practices. Additionally, investment is needed in explicit training in self-care practices, particularly related to the academic/professional domain.

Taken together, our results provide new insights into the interactions between higher education students’ SEC, their self-care practices, and their personal and academic well-being. These findings constitute a significant contribution to the field, shedding light on new avenues for promoting and sustaining the well-being of higher education students.

## Figures and Tables

**Table 1:** Descriptive statistics (Mean and Standard deviation) and correlations (Pearson *r*) between students’ SEC, self-care practices, and personal and academic well-being (*N* = 204).

Variables	*M* (*SD*)	1.	2.	3.	4.	5.	6.	7.	8.	9.
1. Self-awareness	7.46 (1.34)	_								
2. Self-regulation	6.36 (1.50)	0.63	_							
3. Positive relationship skills	6.93 (1.32)	0.63	0.60	_						
4. Conflict management	7.18 (1.14)	0.49	0.55	0.58	_					
5. Responsible decision making	7.03 (1.35)	0.57	0.64	0.67	0.67	_				
6. Personal self-care	3.46 (0.50)	0.31	0.43	0.42	0.24	0.32	_			
7. Academic self-care	3.35 (0.53)	0.32	0.51	0.48	0.28	0.37	0.59	_		
8. Personal well-being	2.58 (0.95)	0.34	0.64	0.43	0.36	0.41	0.57	0.61	_	
9. Academic well-being	3.56 (1.40)	0.27	0.61	0.38	0.37	0.37	0.41	0.50	0.70	_

*Note*. All correlations were statistically significant at *p* < 0.01.

**Table 2:** Component Paths (a and b paths) for Personal Well-being.

Path	*β*	SE	*p*	Interpretation
**a paths (SEC → Mediators)**				
Self-awareness → Personal self-care	0.002	0.034	0.984	Non-significant
Self-awareness → Academic self-care	−0.110	0.036	0.224	Non-significant
Self-regulation → Personal self-care	0.334	0.032	<0.001	Strong effect
Self-regulation → Academic self-care	0.454	0.034	<0.001	Strong effect
Positive relationships → Personal self-care	0.279	0.043	0.014	Moderate effect
Positive relationships → Academic self-care	0.339	0.054	0.011	Moderate effect
**b paths (Mediators → Personal well-being)**				
Personal self-care → Personal well-being	0.227	0.132	0.001	Moderate effect
Academic self-care → Personal well-being	0.273	0.133	<0.001	Strong effect

*Note*. The arrow (→) indicates the direction of the hypothesized path between variables in the mediation model. *β* = standardized regression coefficient; SE = standard error; *p* = significance level. SEC = Social and Emotional Competence.

**Table 3:** Standardized Indirect, Direct, and Total Effects of Social and Emotional Competencies on Personal Well-being.

Variable	Indirect via Personal SC	Indirect via Academic SC	Total Indirect	Direct Effect	Total Effect
Self-awareness	0.004 [−0.036, 0.028]	−0.030 [−0.068, 0.010]	−0.030 [−0.104, 0.038]	−0.112 [−0.211, 0.033]	−0.141 [−0.225, 0.016]
Self-regulation	0.076 * [0.016, 0.106]	0.124 * [0.030, 0.145]	0.200 * [0.046, 0.251]	0.479 * [0.208, 0.482]	0.678 * [0.331, 0.732]
Positive relationship skills	0.063 [−0.008, 0.109]	0.092 * [0.019, 0.143]	0.155 * [0.011, 0.252]	−0.049 [−0.152, 0.079]	0.107 [−0.058, 0.228]
Conflict management	−0.022 [−0.068, 0.010]	−0.026 [−0.067, 0.008]	−0.048 [−0.135, 0.018]	0.067 [−0.077, 0.167]	0.019 [−0.113, 0.158]
Responsible decision making	0.002 [−0.036, 0.033]	−0.005 [−0.044, 0.033]	−0.005 [−0.080, 0.066]	−0.017 [−0.109, 0.098]	−0.022 [−0.124, 0.112]

*Note*. SC = Self-Care. Values are standardized coefficients (*β*) with 95% confidence intervals in brackets. * *p* < 0.05.

**Table 4:** Component Paths (a and b paths) for Academic Well-being.

Path	*β*	SE	*p*	Interpretation
**a paths (SEC → Mediators)**				
Self-awareness → Academic self-care	−0.110	0.033	0.178	Non-significant
Self-regulation → Personal self-care	0.332	0.030	<0.001	Strong predictor
Self-regulation → Academic self-care	0.450	0.030	<0.001	Strong predictor
Positive relationships → Personal self-care	0.284	0.036	0.003	Significant predictor
Positive relationships → Academic self-care	0.350	0.036	<0.001	Strong predictor
**b paths (Mediators → Academic well-being)**				
Academic self-care → Academic well-being	0.208	0.189	0.004	Significant
Personal self-care → Academic well-being	0.108	0.189	0.109	Non-significant

*Note*. The arrow (→) indicates the direction of the hypothesized path between variables in the mediation model. *β* = standardized regression coefficient; SE = standard error; *p* = significance level. SEC = Social and Emotional Competence.

**Table 5:** Standardized Indirect, Direct, and Total Effects of Social and Emotional Competencies on Academic Well-being.

Variable	Indirect via Personal SC	Indirect via Academic SC	Total Indirect	Direct Effect	Total Effect
Self-awareness	0.002 [−0.019, 0.020]	−0.023 [−0.063, 0.015]	−0.023 [−0.071, 0.025]	−0.198 * [−0.363, −0.055]	−0.222 * [−0.394, −0.075]
Self-regulation	0.036 [−0.011, 0.078]	0.094 * [0.020, 0.155]	0.130 * [0.035, 0.225]	0.561 * [0.374, 0.680]	0.692 * [0.501, 0.798]
Positive relationship skills	0.031 [−0.013, 0.078]	0.073 * [0.013, 0.143]	0.104 * [0.022, 0.186]	−0.025 [−0.204, 0.151]	0.078 [−0.094, 0.262]
Conflict management	−0.010 [−0.039, 0.015]	−0.018 [−0.067, 0.021]	−0.028 [−0.091, 0.035]	0.125 [−0.024, 0.335]	0.096 [−0.067, 0.306]
Responsible decision making	−0.004 [−0.022, 0.022]	−0.006 [−0.046, 0.034]	−0.006 [−0.058, 0.046]	−0.062 [−0.238, 0.108]	−0.068 [−0.252, 0.110]

*Note*. SC = Self-Care. Values are standardized coefficients (*β*) with 95% confidence intervals in brackets. * *p* < 0.05.

## Data Availability

The datasets generated and analyzed during this study can be found in the Open Science Framework repository at Oliveira, S. et al. Dataset of the paper “Well-being at the university: The contribution of social and emotional competence and self-care practices as seen by students”. OSF, 17 February 2026. Web. https://doi.org/10.17605/OSF.IO/R3XFY.

## Supplementary Materials

The following supporting information can be downloaded at: https://www.mdpi.com/article/10.3390/bs16071107/s1, Table S1: Category system with an extended summary of quotations.

## Abbreviations

The following abbreviations are used in this manuscript:

CI	Confidence Interval
GLM	Generalized Linear Model
ICC	Intra-class Correlation Coefficient
MHC-SF	Mental Health Continuum—Short Form
SCPS	Self-care Practices Scale
SEC	Social and Emotional Competence
SECAB-A(S)	Social and Emotional Competence Assessment Battery for Adults—Students Survey
SEL	Social and Emotional Learning
UWES-S	Utrecht Work Engagement Scale for Students
