# Supplementary material for: Well-Being at the University: The Contribution of Social and Emotional Competence and Self-Care Practices as Seen by Students
Source: Behav Sci (Basel). 2026 Jul 3;16(7):1107. doi: 10.3390/bs16071107 (PMC13406037; doi:10.3390/bs16071107)
Supplement: Supplementary file 1 [file behavsci-16-01107-s001.zip › behavsci-4331381-supplementary.pdf]

behavioral sciences

MDPI

Table S1. Category system with an extended summary of quotations ($N = 16$, Total recording units = 480).

Theme	Categories (*f*; %)	Subcategories—Operational definition of indicators (*f*; %)	*% cases*	Illustrative quotations
**Challenges to university adaptation**	**Socioemotional** ($f = 51$; 64.6%)	**Emotional**—Includes demotivation, stress, expectation management, social comparison, maladaptive coping ($f = 19$; 35.8%)	75.0%	"I've always been a bit of a perfectionist, so it was a little hard to adjust to the idea that I won't get perfect grades in my program and I won't perform perfectly in the field I've always dreamed of." (P01)<br>"I had to learn to deal with different emotions—frustration, anxiety, and fear." (P05)<br>"These adaptations have been very difficult for me to cope with; I start to doubt whether I'll be able to get through this, thinking I don't have the ability, that I lack the skills, that it's going to be hard." (P08)
		**Social and relational**—Includes building new relationships and maintaining previous ones ($f = 19$; 35.8%)	68.8%	"My college friends—I feel like there's a certain distance between us. For example, all my friends live in different places." (P07)<br>"So it took a little while to adjust emotionally, too—I didn't know anyone, and it took me a while to find people I could relate to" (P10)
		**Personal organization**—Includes increased autonomy, time management, and study methods ($f = 13$; 25.4%)	56.3%	"I had to learn to manage my time better, both for personal matters and for academic ones." (P05)<br>"It seems like there's always a lot to do." (P12)

				"I often stay up all night because I have to get things done. Why? Because during the day I got distracted by other things I shouldn't have." (P13)
	**Contextual and logistical** (*f* = 17; 21.5%)—Includes family context, new environment, distance from home, finances, transportation logistics.		68.8%	"I'm far from my family, I'm far from people in general, I live alone, so I have to take care of myself, right?" (P01)<br>"I have to take public transportation, all that logistical planning, all those things, that's what has changed the most in my life." (P07)<br>"Since I was a student living away from home, adjusting was a little harder for me, because I didn't have my support network where I was." (P08)
	**Academic** (*f* = 11; 13.9%)—Includes assessments, academic demands, schedules, and specific course-related difficulties.		50.0%	"The pace is different from high school. So, there's a lot going on at once. Sometimes I feel like I can't keep up, and that's a little frustrating." (P02)<br>"I think I was less prepared to handle the workload we were going to have and the demands of college, studying, and all that." (P07)<br>"There's a downside to it, right? In terms of well-being, it's the grades and the pressure we feel." (P15)
**Social and Emotional Competencies**	**Intrapersonal** (*f* = 116; 60.1%)	**Self-awareness**—Includes adequate self-perception, emotional awareness and optimism (*f* = 62; 53.4%)	87.5%	"To realize deep down what exactly was bothering me." (P01)<br>"I think I've realized that I might actually be much more capable of doing things on my own and managing my life by myself than I ever thought I would be." (P04)<br>"I've learned a lot, I've grown a lot, and I've gotten to know myself quite well." (P12)

	**Self-regulation**—Includes organizational skills, goal setting and pursuit, flexibility, emotional and behavioral regulation, self-efficacy (*f* = 54; 46.6%)	81.3%	"Set priorities, set goals, and manage my time well—and not forget to be flexible with the schedule I set for myself." (P01)<br>"Today I'm spending these two hours with friends, but in return, I'll have to fit these two hours of studying into some other part of the day or week. So I try to strike a balance." (P02)<br>"It's about letting myself experience that moment, maybe just being there alone, letting loose a little, maybe sometimes it's necessary to cry or scream, to let out a little of that anger, maybe from the frustration of something, from that challenge, and then manage those emotions." (P12)<br>"If it's a stressful situation, I usually try to calm myself down, take a deep breath, and take it easy." (P14)
**Interpersonal** (*f* = 72; 37.3%)	**Social awareness**—Includes understanding social norms, empathy, recognition of others' emotions, perspective-taking, and identification of available resources and support (*f* = 24; 33.3%)	68.8%	"I think I've always been very observant of the people around me (…), I've always paid close attention to those little details, and so I think I've always been good at picking up on other people's feelings." (P01)<br>"I need to take a step back, I need to breathe, I need to understand that this is their reality." (P08)<br>"Fortunately, I have a support system—a very large network of people who support me. Both my parents and my boyfriend are very important to me." (P09)
	**Relationship skills**—Includes competences used to establish and maintain relationships and to manage	87.5%	"Let's work together to get the job done. It's all about talking openly with people and not jumping to conclusions without talking to them first." (P02)

		conflict, such as communication skills, cooperation, conflict management skills, seeking and providing help (*f* = 48; 66.7%)		"I've always tried to keep that mindset of 'I'm going to talk to people, I'm going to try to get to know them, because I'm here and I don't know anyone' so I tried to approach things with a more open mind, talking to people and getting to know them, and that's it—it's been a good experience." (P04)<br>"Spending time with these people and talking to the ones I care about most, so they know I love them and that they're important to me." (P10)
	**Responsible decision-making** (*f* = 5; 2.6%)—Includes behavioral evaluation and reflection, problem identification, analysis of solutions, and problem-solving.		25.0%	"To acknowledge that things aren't going well and at the same time say, 'Okay, if they're not going well, I'm going to have to take care of myself, because otherwise things will continue to go wrong.'" (P03)<br>"I think it's a matter of putting things into perspective, figuring out what the priorities are, and making decisions, isn't it?" (P04)
**Self-care**	**Personal self-care practices** (*f* = 195; 93.7%)	**Psychological/Emotional** (*f* = 94; 48.2%)—Includes self-development activities, leisure, emotional management, and restorative breaks.	100%	"When I'm alone, I also like to do journaling." (P01)<br>"Sometimes I start a TV show (…) or I go to bed, so it's that moment to unwind before actually resting." (P02)<br>"Psychotherapy, because it's something I really prioritize." (P05)<br>"Listening to music I like, that helps me relax too." (P06)<br>"Taking care of my appearance, basically, wearing something nice, something that makes me feel good because I feel that dressing well helps me maintain a positive self–image." (P08)<br>"I've also been doing some puzzles." (P11)

				“Sometimes it's good to just be alone, to have a moment to myself.” (P12)<br>“Getting involved in activities I enjoy doing.” (P14)<br>“Spending time with my dog. For me, it's a way of taking care of myself, you know?” (P 15)
		**Social** (*f* = 63; 32.3%)—Includes social interaction, relational rituals, intimacy, and affective validation.	100%	“Try to spend quality time with [my friends], talk, do something that’s fun, relaxing, and takes our minds off our responsibilities.” (P04)<br>“Talking to someone about how I feel is really important.” (P08)<br>“Spending time with people I care about or even meeting new people.” (P13)
		**Physical** (*f* = 38; 19.5%)—Includes nutrition, sleep, body care, physical activity.	75.0%	“I drink a lot of water.” (P01)<br>“A moment of self-care focusing on my skin, hair, and all that. It helps me go to bed feeling more relaxed.” (P03)<br>“Maintaining a good sleep routine.” (P04)<br>“Exercise and the gym are very important to me.” (P09)<br>“Cooking is also something that helps me take care of myself.” (P16)
	**Academic self-care practices** (*f* = 13; 6.3%)—Includes attention to the academic role, academic development, time and workload management, taking breaks, workspace organization, and restorative/recovery activities.		56.3%	“When I'm studying, my phone stays out of sight—preferably even out of the room where I'm studying.” (P02)<br>“I think what I do most often is take more frequent breaks to really ease up on the intensity of studying and sometimes the intensity of the emotions that come with studying.” (P03)

	"My relationships with colleagues offer me more practical support. For example, when it comes to questions or even during exam season, we help each other out with more specific issues." (P05)<br>"My dad and I, for example, we do study sessions. He's working and I'm studying, sometimes on a video call. It's a bit unusual, but it's a way for both of us to focus a little better." (P09)<br>"Learn that if nothing is getting through anymore, take a break. Clear your head and come back in a little while." (P16)

Note: f = frequency of recording units coded in the (sub-)category; % cases = percentage of cases in which at least one recording unit was coded.
